# Interinstitutional collaboration to promote public health research in Ecuador: a story of success from the National Institute of Public Health in Ecuador

**DOI:** 10.3389/fpubh.2026.1828467

**Published:** 2026-08-24

**Authors:** Solon Alberto Orlando, Alfredo Bruno, Greta Franco-Sotomayor, Miguel Angel García-Bereguiain

**Affiliations:** 1 Instituto Nacional de Salud Pública e Investigación, Guayaquil, Ecuador; 2 Universidad Espíritu Santo, Guayaquil, Ecuador; 3 Universidad Agraria de Ecuador, Guayaquil, Ecuador; 4 Universidad Católica Santiago de Guayaquil, Guayaquil, Ecuador; 5 One Health Research Group, Universidad de Las Américas, Quito, Ecuador

**Keywords:** academia, Ecuador, One Health, public health, public health policy

## Introduction

Back in the fall of 2016, we started a collaboration between the “Instituto Nacional de Salud Pública e Investigación” (INSPI) and different Ecuadorian public and private universities in Quito and Guayaquil, initially including the “Universidad Agraria de Ecuador” and “Escuela Superior Politécnica del Litoral, and later “Universidad de Las Américas,” “Universidad Ecotec” and “Universidad Espiritu Santo.” We were able to put together a group of scientists with different professional expertise from both the public health sector, such as INSPI, and academia. Moreover, the team included professionals from different disciplines, including veterinarians, physicians, and molecular biologists. This multidisciplinary team shared a common interest in the emerging concept of One Health more than a decade ago.

## Context

Nevertheless, establishing this collaboration was not easy. Scientists from INSPI were initially cautious about this type of collaboration due to the traditional academic view of INSPI as primarily a provider of infectious diseases samples, which generated frustration among many INSPI researchers and reluctance to collaborate with universities. In this sense, from the very beginning, collaborators from INSPI made it clear that the success and sustainability of this inter-institutional collaboration would depend on a clear “win-win” strategy in which all the stakeholders involved would benefit. The starting point was to identify our strengths and weaknesses and to seek a truly synergistic collaboration.

## Institutional collaboration model

This new interinstitutional collaboration aimed to move beyond the previous model, in which INSPI provided access to samples and data and was included only as a co-author or in the acknowledgments of related scientific publications. The new model would promote genuine leadership by INSPI in public health research projects. As a public institution in a low-and-middle-income country (LMIC), the main weakness of INSPI was its budget constraint on research, as its primary activity is epidemiological surveillance. At the same time, the strengths of INSPI included a well-trained staff capable of diagnosing infectious diseases according to national and international quality standards, collections of data and samples accumulated through years of surveillance of the main infectious diseases affecting Ecuador, and extensive knowledge in the diagnosis of infectious diseases in the context of LMICs. On the other hand, academia would facilitate access to research funding through universities (especially private universities such as the Universidad de Las Américas) and PhD training programs that would improve the research capacity of INSPI collaborators.

Thus far, this institutional collaboration model has bypassed the main challenge of insufficient and sustained research funding for public institutions in LMICs such as Ecuador. Funding from private universities has been crucial for providing the supplies needed to support the research projects associated with this initiative. However, the involvement of INSPI staff has ensured sustainable labor costs for this research project.

## Impact on research capacity and PhD training

Almost 10 years have passed since we started this collaboration, and important milestones have been reached. Three leading scientists from INSPI joined a PhD program in Public Health, working on fundamental research areas of interest at INSPI such as tuberculosis, respiratory viruses, leptospirosis, and other zoonotic diseases. One of them already earned a PhD degree in December 2024, while the other two are in the pipeline for PhD dissertations in 2027. This pilot experience in PhD training has laid the groundwork for sustainable research training for INSPI staff, with new PhDs affiliated with INSPI mentoring new trainees through extended partnerships with Ecuadorian and international universities.

This intense research activity has also been reflected in an extensive record of scientific publications since 2019 (some of which are detailed in the references) (1), including 14 publications on the diagnosis, epidemiology, and zoonotic risks of tuberculosis and other mycobacteria (1–5); 15 publications on the diagnosis, epidemiology, genomics, and zoonotic risks of respiratory viruses, including influenza viruses and SARS-CoV-2 (1, 6–10); and 16 publications on the diagnosis and epidemiology of several zoonotic diseases in humans and animals, such as leptospirosis, brucellosis, and bordetellosis (1, 11–15). These scientific contributions have positioned INSPI as one of the top Ecuadorian institutions and a regional reference center for public health research.

## Transfer of knowledge

Beyond PhD training and publications, it is important to highlight the knowledge transfer associated with this initiative. New diagnostic protocols have been developed as part of these research projects, and the INSPI PhD trainees have been leading the transition from more traditional methods to state-of-the-art molecular diagnostics for tuberculosis and other mycobacteria, influenza and other respiratory viruses, arboviruses, leptospirosis and other emerging pathogens, while also improving genomic surveillance in Ecuador. So far, this interinstitutional collaboration has also enabled truly translational research with direct transfer of bench results to improvements in diagnosis and surveillance of infectious diseases in Ecuador.

## One Health perspective

Finally, as we mentioned in the first paragraph, this teamwork was established by physicians, veterinarians, and molecular biologists with a common interest in the emerging concept of One Health more than 10 years ago. The integration of human, animal, and environmental health, promoted by the One Health concept, is especially crucial for controlling and preventing infectious diseases in LMICs such as Ecuador. Moreover, since its establishment, INSPI has been the national reference center for the diagnosis and surveillance of infectious diseases in Ecuador. Therefore, the One Health perspective has inspired this entire research journey. Not only have new protocols been developed to improve infectious disease diagnosis and surveillance in humans, but also this research team has conducted multiple studies to identify animal reservoirs of multiple zoonotic diseases, such as tuberculosis, brucellosis, bartonellosis, leptospirosis, or influenza (1–15).

## Future directions and conclusion

Our collaboration is expected to continue in the years to come for the advancement of public health research in Ecuador. The main conclusion of this opinion piece is clear: a “win-win” strategy based on fair interinstitutional collaboration is fundamental for a sustained and solid partnership between INSPI and academia. New PhD trainees will continue to improve research capacities within INSPI. New pathogens will be studied. Moreover, our pilot experience in animal health research should inspire the development of a national surveillance program for infectious diseases from a One Health perspective, involving INSPI (Ministry of Health), Agrocalidad (the National Reference Veterinary Laboratory of the Ministry of Agriculture and Livestock), and the Ministry of Environment in Ecuador.
